# Predictors of Two Months Culture Conversion in Multidrug-Resistant Tuberculosis: Findings from a Retrospective Cohort Study

**DOI:** 10.1371/journal.pone.0093206

**Published:** 2014-04-04

**Authors:** Anila Basit, Nafees Ahmad, Amer Hayat Khan, Arshad Javaid, Syed Azhar Syed Sulaiman, Afsar Khan Afridi, Azreen Syazril Adnan, Israr ul Haq, Syed Saleem Shah, Ahmed Ahadi, Izaz Ahmad

**Affiliations:** 1 Post Graduate Medical Institute, Peshawar, Pakistan; 2 Discipline of Clinical Pharmacy, School of Pharmaceutical Sciences, Universiti Sains Malaysia, Pulau Pinang, Malaysia; 3 Department of Pulmonology, Lady Reading Hospital, Peshawar, Pakistan; 4 Programmatic Management Unit of Drug Resistant TB, Lady Reading Hospital, Peshawar, Pakistan; 5 Chronic Kidney Disease (CKD) Resource Center, School of Medical Sciences, Universiti Sains Malaysia, Kota Bharu, Kelantan, Malaysia; 6 Department of Pediatrics, Saidu Medical College, Saidu Sharif Swat, Pakistan; 7 Khyber Medical University, Peshawar, Pakistan; 8 Department of Pharmacy, University of Balochistan, Quetta, Pakistan; 9 Department of Microbiology, Quaid-e- Azam University, Islamabad, Pakistan; University of Iowa Carver College of Medicine, United States of America

## Abstract

**Background:**

Various studies have reported culture conversion at two months as a predictor of successful treatment outcome in multidrug-resistant tuberculosis (MDR-TB).

**Objectives:**

The present study was conducted with the aim to evaluate the rate and predictors of culture conversion at two months in MDR-TB patients.

**Methods:**

All confirmed pulmonary MDR-TB patients enrolled for treatment at Lady Reading Hospital Peshawar, Pakistan from 1 January to 31 December 2012 and met the inclusion criteria were reviewed retrospectively. Rate and predictors of culture conversion at two months were evaluated.

**Results:**

Eighty seven (53.4%) out of 163 patients achieved culture conversion at two months. In a multivariate analysis lung cavitation at baseline chest X-ray (P = 0.006, OR = 0.349), resistance to ofloxacin (P = 0.041, OR = 0.193) and streptomycin (P = 0.017, OR = 0.295) had statistically significant (P<0.05) negative association with culture conversion at two months.

**Conclusion:**

A reasonable proportion of patients achieved culture conversion at two months. Factors negatively associated with culture conversion at two months can be easily identified either before diagnosis or early in the course of MDR-TB treatment. This may help in better care of individual patients by identifying them early and treating them vigorously.

## Introduction

Multi drug resistant tuberculosis (MDR-TB) involves prolonged treatment, expensive and toxic regimens, more potent sources of infection for others, and reflects higher rates of treatment failure as compared to drug susceptible TB [1]. Culture conversion, defined as “two consecutive negative sputum cultures taken at least 30 days apart following an initial positive culture” is considered a reliable indicator of non-infectiousness [2], [3], is used as a guide for therapy and a cardinal interim indicator for predicting treatment success in both susceptible as well as drug resistant TB [4], [5]. In MDR-TB management the use of injectable, shift from intensive to continuation phase and defining treatment outcomes are mainly based on microbiological status of patients’ sputum culture. Early culture conversion has been widely reported as a predictor of treatment success in both susceptible and drug resistant TB [6]–[10]. Culture conversion at two months has widely been reported as a strong predictor and early indicator of treatment success in drug susceptible TB [9]–[11]. Similarly, better treatment outcomes have been observed in MDR-TB patients who achieved culture conversion at two months and vice versa. In a study conducted in Hong Kong negative culture at two months MDR-TB treatment was 100% predictive of cure [7]. Better treatment outcomes have been observed in MDR-TB patients who achieved culture conversion at two months in a study conducted in Dominican Republic [8]. On the other hand poor treatment outcomes have been reported in MDR-TB patients who failed to achieve culture conversion at two months [10]. Identification of potential factors associated with culture conversion at two months in MDR-TB may help in stratification of patients and provision of an opportunity for clinicians to identify in advance and vigorously treat the patients who are at risk of developing poor outcomes. Although few studies have evaluated time to and factors associated with early culture conversion in MDR-TB [1], [4], [6], to the best of our knowledge no study has evaluated predictors of culture conversion at two months in MDR-TB. Thus the present study was undertaken with the aim to evaluate the rate and predictors of sputum culture conversion at two months in pulmonary MDR-TB patients.

## Methods

### Study Design and Settings

This was a retrospective cohort study conducted at Programmatic Management Unit of MDR-TB, Lady Reading Hospital (LRH) Peshawar, Pakistan. All confirmed pulmonary MDR-TB patients [patients having a positive sputum culture for *M. tuberculosis* with in-vitro resistance to at least isoniazid (INH, H) and rifampicin (Rif, R)] who were consecutively enrolled for treatment at the study site from 1 January 2012 to 31 December 2012 and had a follow up of at least three visits were included in the study. Patients with negative culture at the time of treatment initiation, contaminated second and/or third culture and culture not done on second and third months of treatment were excluded. At the time of the study LRH was the only center in Khyber Pukhtoonkhwa (one of the four provinces of Pakistan) where MDR-TB patients were treated.

All patients enrolled in the study were treated on an ambulatory based strategy and were seen monthly by a team of clinicians. Patients presented with the signs and symptoms and treatment history of TB were evaluated with two sputum samples for Acid Fast Bacilli (AFB) by sputum smear microscopy using Ziehl Neelsen staining method. In order to increase the likelihood of providing appropriate initial therapy right from the beginning of the treatment, rapid drug susceptibility testing (DST) for detecting rifampicin resistance by using Xpert MTB/Rif (*Mycobacterium tuberculosis*/rifampicin) was performed for most patients. Upon positive sputum smear microscopy and detection of rifampicin resistance by MTB/Rif Xpert, patients were placed on standardized treatment regimen of second line drugs. Standardized treatment regimen was formulated on the basis of patients’ history of TB treatment and confirmed contact with MDR-TB patient and was continued until the results of DST. Patients were then shifted to individualized regimens once DST results were obtained. Sputum smear and cultures were performed at baseline and monthly follow up visits during the course of the treatment. For sputum culture and DST, samples collected were sent to National Reference Laboratory (NRL) Islamabad of National Tuberculosis Program (NTP) (a BSL3 laboratory with proficiency testing approved by Supra National Lab in Belgium) and Agha Khan University Hospital Laboratory respectively. DST was performed on all culture positive isolates against first line [isoniazid (INH, H), rifampicin (Rif, R), pyrazinamide (PZA, Z), ethambutol (E) and streptomycin (S)] and second line anti TB drugs [(amikacin (Am), kanamycin (Km), capreomycin (Cm), ofloxacin (Ofx), ethionamide (Eto), cycloserine (Cs) and para-amino salicylic acid (PAS)]. Medication adherence was monitored by trained treatment supporters and directly observed therapy facilitators. Patients were psychologically evaluated and personalized counseling was provided to them on monthly follow up visits.

A purpose developed validated data collection form was used to collect patients’ demographic, clinical and microbiological data. Demographic data included sex, age, weight, comorbidities, area of residence, close contacts TB status, and smoking, drinking and intravenous drugs usestatus. Clinical data included history and outcome of previous TB treatment, previous use of second line drugs and radiological findings at baseline chest X-ray. Microbiological data included sputum smear grading at baseline and DST results at the baseline visit and microbiological culture status at monthly follow up visits.

### Statistical Analysis

Data was analyzed by using SPSS 16. Categorical variables were presented as percentages and frequencies and means and standard deviations were calculated for continuous variables. Chi-squared test was used to observe significance between categorical variables. Multivariate analysis was used to obtain a final model describing the significant independent predictors of culture conversion at two months. The results of multivariate analysis were presented as beta, standard error, *P*-value, adjusted odds ratio and 95% confidence interval. The fit of the model was assessed by Hosmer Lemeshow and overall classification percentage. Significance of the statistical tests was taken at a *P*-value of <0.05.

### Ethical Approval

This study was approved by the ethical research committee of Lady Reading Hospital Peshawar. Written consent for use of clinical record was taken from patients who were alive and visiting the study site for their treatment prior to the beginning of the study. All patients’ record/information was anonymized and de-identified prior to analysis.

## Results

During the study period 180 pulmonary MDR-TB patients were enrolled for treatment at the study site. A total of 163 met the inclusion criteria and were included in the final analysis. Two patients were excluded because of negative culture at the time of treatment initiation; 4 because of death in the initial three months of therapy and 11 because of contaminated culture results at either 2^nd^ and/or 3^rd^ month of their treatment. Patients’ demographic and clinical characteristics included in the final analysis are given in Table 1. Sputum isolates from one patient was resistant to two drugs (HR), 11 to three drugs (8 to HRZ and 3 to HRE), 17 to four drugs (6 to HRZ+Ofx, 5 to HRZE, 5 to HRZS and1 to HRE+Ofx), 66 to five drugs (43 to HRZES, 19 to HRZE+Ofx, 2 to HRZS+Ofx, 2 to HRES+Ofx), 61 to six drugs (56 to HRZES+Ofx, 2 to HRZES+Cs, 2 to HRZES+ Eto and 1 to HRZES+Cm), and 7 to seven drugs (HRZES+Ofx+Eto).

**Table 1:** Patients’ demographics and clinical characteristics.

Variables	Means±SD	No. (%)
**Gender**		
Male		80 (49.1)
Female		83 (50.9)
**Age (years)**	31.7±14.7	
≤40		117 (71.8)
>40		46 (28.2)
**Weight (Kg)**	45.1±10.6	
≤40		64 (39.3)
41–60		87 (53.4)
>60		12 (7.4)
**Residence**		
Urban		60 (36.8)
Rural		103 (63.2)
**Marital status**		
Married		89 (54.6)
Unmarried		69 (42.3)
Widow		5 (3.1)
**Smoking**		
Active+ Ex-smokers		22 (13.4)
Non-smokers		141 (86.6)
**Patients contact status**		
No TB		103 (63.2)
Drug susceptible TB		35 (21.5)
PDR-TB		2 (1.2)
MDR-TB		23 (14.1)
**Comorbidity**		
Yes		24 (14.3)
No		139 (85.3)
**History of SLD use**		
Yes		11 (6.7)
No		152 (93.3)
**Registration group**		
New		18 (11.0)
Relapse		14 (8.6)
Category 1 failure		45 (27.6)
Category II failure		63 (38.7)
Others		23 (14.1)
**Normal hemoglobin level at baseline visit**(male ≥13.5 gm/dl, female ≥12 gm/dl)		
Yes		44 (27.0)
No		119 (73.0)
**Lung cavitation at baseline chest X-ray**		
Yes		65 (39.9)
No		98 (60.1)
**Smear grading at baseline**		
+3 (>9 AFB/HPF)		57 (35.0)
+2 (1–9 AFB/HPF)		36 (22.1)
+1 (10–99 AFB/100 HPF)		57 (35.0)
Scanty (1–9 AFB/100 HPF)		1 (0.4)
Negative		12 (7.4)
**Resistance to drugs at treatment initiation**		
2–4 drugs		29 (17.8)
5–6 drugs		127 (77.9)
7 drugs		7 (4.3)
**Resistant to second line drugs**		
Yes		94 (57.7)
No		69 (42.3)
**Resistance to ofloxacin**		
Yes		93 (57.1)
No		70 (49.2)
**Resistance to cycloserine**		
Yes		2 (1.2)
No		161 (98.8)
**Resistance to ethionamide**		
Yes		10 (6.1)
No		153 (93.9)
**Resistance to streptomycin**		
Yes		120 (73.6)
No		43 (26.4)
**Resistance to ethambutol**		
Yes		139 (85.3)
No		24 (14.7)
**Resistance to pyrazinamide**		
Yes		156 (95.7)
No		7 (4.3)
**Resistance to kanamycin**		
Yes		1 (0.6)
No		162 (99.4)
**Delay in MDR-TB treatment initiation**		
<2 months		132 (81.0)
≥2 months		31 (19.0)

AFB, acid-fast bacilli; MDR, multi drug resistance PDR, poly-drug resistance; HPF, high power field; SLD, second-line drugs; TB, tuberculosis.

The baseline regimens received for two months or more are as follows: 93 (57.1%) patients received Am+Z+Eto+Cs+Lfx (levofloxacin), and Vitamin B6, 24 (14.7%) received Am+Z+Eto+Cs+Lfx+PAS and Vitamin B6, 41 (25.2%) received Km+Z+Eto+Cs+Lfx and Vitamin B6 and 5 (3.1%) received Km+Z+Eto+Cs+Lfx+PAS and Vitamin B6. Para-amino salicylic acid was added to the baseline regimens of those patients in whom either treatment was initiated after DST results and had resistance to flouroquniolones or who had known flouroquniolone resistant MDR-TB contact or history of use of second line anti TB drugs.

Eighty seven (53.4%) patients achieved culture conversion at two months. On univariate analysis, culture conversion at two months had statistically significant negative association with lung cavitation at baseline chest X-ray (OR = 0.500, P = 0.033), resistance to second line drugs (OR = 0.310, P<0.001), streptomycin (OR = 0.339, P = 0.005), ethionamide (OR = 0.087, P = 0.022), and ofloxacin (OR = 0.296, P<0.001), and had statistically significant positive association (OR = 3.336, P = 0.010) with resistance to 2–4 drugs at treatment initiation (Table 2).

**Table 2:** Univariate analysis of predictors of sputum culture conversion at two months.

Variables	Sputum culture conversion at the end of two months (n, %) Yes No		95% CI	Odds ratio	P-value
**Lung cavitation at baseline chest X-ray**					
Cavitation	28 (43.1)	37 (56.5)	0.265–0.945	0.500	0.033
No cavitation	59 (60.2)	39 (39.8)	Referent		
**Resistance to second line drugs**					
Yes	39 (41.5)	55 (58.5)	0.310–0.161	0.310	<0.001
No	48 (69.6)	21 (30.4)	Referent		
**Resistance to drugs at treatment initiation**					
2–4 drugs	22 (75.9)	7 (24.1)	1.336–8.334	3.336	0.010
7 drugs	0 (0.0)	7 (100.0)	Referent		
**Resistance to streptomycin**					
Yes	56 (46.7)	64 (53.3)	0.159–0.722	0.339	0.005
No	31 (72.1)	12 (27.9)	Referent		
**Resistance to ethionamide**					
Yes	1 (10.0)	9 (90.0)	0.011–0.700	0.087	0.022
No	86 (56.2)	67 (43.8)	Referent		
**Resistance to ofloxacin**					
Yes	38 (40.9)	55 (59.1)	0.153–0.571	0.296	<0.001
No	49 (70.0)	21 (30.0)	Referent		

Note: Only statistically significant results are given in the table.

CI, confidence interval; TB, tuberculosis.

In multivariate analysis lung cavitation at baseline chest X-ray (OR = 0.349, P = 0.006), resistance to ofloxacin (OR = 0.193, P = 0.041) and streptomycin (OR = 0.295, P = 0.017) had statistically negative association with culture conversion at two months (Table 3). This model fit was based on a non-significant Hosmer and Lemeshow test (P = 0.322) and overall percentage of 69.3% from the classification table.

**Table 3:** Multivariate analysis of predictors of sputum culture conversion at two months.

Variables	B	SE	95% CI	Odds ratio	P-value
**Lung cavitation at baseline chest X-ray**	−1.053	0.382	0.165–0.737	0.349	0.006
**Resistance to ofloxacin**	−1.646	0.805	0.40–0.933	0.193	0.041
**Resistance to streptomycin**	−1.220	0.509	0.109–0.801	0.295	0.017

Note: Only statistically significant results are given in the table.

B, beta; CI, confidence interval; SE, standard error.

## Discussion

In the present study more than half (53.4%) of MDR-TB patients achieved sputum culture conversion at two months. This was comparable to the findings of studies conducted in India and Dominican Republic, where 46% and 48.8% of MDR-TB patients respectively achieved culture conversion at two months of treatment [8], [12], but was better than culture conversion rate of 30% at two months observed in a study conducted in Latvia [6]. In contrast to our finding, Joseph *et al* has reported a much higher percentage (82%) of MDR-TB patients who achieved culture conversion at two months in a study in conducted in India [13]. But the results of the Joseph *et al.* study should be interpreted with notable limitation of small number of patients enrolled (n = 38) in the study [13]. Despite a high rate of drug resistance (particularly to ofloxacin, 57.1%), the reasonable rate of culture conversion at the two months in our study is encouraging, and indicates the effectiveness of MDR-TB management at the study site. One of the possible reasons for better rate of early culture conversion could be the provision of appropriate initial therapy right from the beginning of the treatment. In the present study upon positive sputum smear microscopy and detection of rifampicin resistance by MTB/Rif Xpert, the majority of patients were placed on standardized treatment regimen of second line drugs right from the beginning rather than waiting for DST results. The presence of trained community treatment supporters for directly observed therapy might also have played a role in achieving fair level of culture conversion at the end of two months of treatment.

Lung cavitation is a well-recognized risk factor of delayed culture conversion and treatment failure in TB [14]–[16]. In multivariate analysis we found a statistically significant negative association between lung cavitation at baseline chest X-ray and culture conversion at two months. As presence of lung cavities decrease penetration and antibacterial activity of drugs [7], negative association between lung cavitation and culture conversion at two months in the present study is not unexpected and is in agreement with studies conducted in Latvia and South Africa [6], [17]. In contrast to our finding, studies conducted in US and Pakistan has found no significant association between lung cavitation at baseline chest X-ray and culture conversion [1], [4]. Resistance to various drugs like fluoroquinolones, kanamycin and pyrazinamide has previously been reported as a predictor of delayed culture conversion in MDR-TB [13], [18]. In our study resistance to ofloxacin and streptomycin achieved the level of significance in multivariate analysis and had negative association with culture conversion at two months. Fluoroquinolones have a significant role in curing MDR-TB patients. Their use is a significant predictor of early culture conversion and correlates with survival in MDR-TB [1], [7]. As fluoroquinolones have fast bactericidal and sterilizing effect [7], negative association between resistance to ofloxacin and culture conversion at two months observed in our study is not astonishing and is line with studies conducted elsewhere [18], [19]. Despite its pivotal role in MDR-TB treatment, high rate of resistance to ofloxacin (57.1%) observed in our patients is alarming and is in compliance with reported increase in fluoroquinolones resistant MDR-TB isolates (17.41% in 2005 to 42.92% in 2009) in Pakistan [20]. The non-prescription sale, over the counter availability and irrational prescription of fluoroquinolones by physicians are major reasons of increase in fluoroquinolones resistant MDR-TB strains in Pakistan [20], [21]. Adoption of more restrictive policies to discourage the use of fluoroquinolones as routine antibiotics especially in the presence of other active drugs can prevent further increase in fluoroquinolones resistance in Pakistan. Another important finding in the present study was negative association between resistance to streptomycin and culture conversion at two months. The reason for this finding is not clear. Although various studies have reported resistance to streptomycin as a predictor of adverse treatment outcomes in extensive-drug resistant (XDR) TB and pre-XDR TB [22], [23], but no study has reported relationship between resistance to streptomycin and delayed culture conversion in MDR-TB [4], [6], [8], [13]. Proportion of streptomycin susceptible patients is a notable difference between the present study and the one conducted by Holtz *et al*. in which no significant association has been observed between resistance to streptomycin and culture conversion. In our study 26.4% were susceptible to streptomycin as compared to only 5.3% in Hotlz *et al*. study [6]. Other studies which have reported no association between resistance to streptomycin and culture conversion lack information on rate of resistance to streptomycin [4], [8], [13]. Further information is needed to confirm this finding.

The notable limitation associated with the present study is its retrospective design and inability to evaluate total culture conversion. At the time of the study, the majority patients included in the analysis were still on treatment due to which we were unable to correlate culture conversion at two months of treatment with treatment outcomes.

### Conclusion

Despite high rate of drug resistance in studied population, an overall fair proportion of patients achieved culture conversion at two months. Large number of patients resistant to ofloxacin in high MDR-TB burden country like Pakistan is alarming. Authorities need to have a closer look at this issue. Factors negatively associated with culture conversion at two months in the present study can be easily identified either before diagnosis or early in the course of MDR-TB therapy. This may help in better care of individual patients by identifying them early and treating them vigorously. Further studies are needed to confirm the finding of negative association between resistance to streptomycin and culture conversion at two months observed in the present study.
